# RETRACTED: Cavalu et al. Characterization of the Anti-Biofilm and Anti-Quorum Sensing Activities of the β-Adrenoreceptor Antagonist Atenolol Against Gram-Negative Bacterial Pathogens. *Int. J. Mol. Sci.* 2022, *23*, 13088

**DOI:** 10.3390/ijms27104196

**Published:** 2026-05-08

**Authors:** Simona Cavalu, Samar S. Elbaramawi, Ahmed G. Eissa, Mohamed F. Radwan, Tarek S. Ibrahim, El-Sayed Khafagy, Bruno Silvester Lopes, Mohamed A. M. Ali, Wael A. H. Hegazy, Mahmoud A. Elfaky

**Affiliations:** 1 Faculty of Medicine and Pharmacy, University of Oradea, P-ta 1 Decembrie 10, 410087 Oradea, Romania; 2 Medicinal Chemistry Department, Faculty of Pharmacy, Zagazig University, Zagazig 44519, Egypt; 3 Department of Pharmaceutical Chemistry, Faculty of Pharmacy, King Abdulaziz University, Jeddah 21589, Saudi Arabia; 4 Department of Pharmaceutics, College of Pharmacy, Prince Sattam Bin Abdulaziz University, Al-kharj 11942, Saudi Arabia; 5 Department of Pharmaceutics and Industrial Pharmacy, Faculty of Pharmacy, Suez Canal University, Ismailia 41522, Egypt; 6 School of Health and Life Sciences, Teesside University, Middlesbrough TS1 3BA, UK; 7 National Horizons Centre, Teesside University, Darlington DL1 1HG, UK; 8 Department of Biology, College of Science, Imam Mohammad Ibn Saud Islamic University, Riyadh 11432, Saudi Arabia; 9 Department of Biochemistry, Faculty of Science, Ain Shams University, Abbassia, Cairo 11566, Egypt; 10 Department of Microbiology and Immunology, Faculty of Pharmacy, Zagazig University, Zagazig 44519, Egypt; 11 Pharmacy Program, Department of Pharmaceutical Sciences, Oman College of Health Sciences, Muscat 113, Oman; 12 Department of Natural Products, Faculty of Pharmacy, King Abdulaziz University, Jeddah 21589, Saudi Arabia; 13 Centre for Artificial Intelligence in Precision Medicines, King Abdulaziz University, Jeddah 21589, Saudi Arabia

The journal retracts the article titled “Characterization of the Anti-Biofilm and Anti-Quorum Sensing Activities of the β-Adrenoreceptor Antagonist Atenolol Against Gram-Negative Bacterial Pathogens” [1], cited above.

Following the publication, concerns were brought to the attention of the Editorial Office regarding the integrity and accuracy of the images and data presented in the published paper [1].

In accordance with the journal’s standard procedures, the Editorial Office and Editorial Board conducted an investigation that identified indications of inappropriate image editing and duplication between this article and a number of previously published works produced by the same authorship group. These concerns relate to the images and data presented in Figure 9. Following further discussions with the authors, the Editorial Board was unable to verify the integrity of the figure in question based on the explanations and supplementary material provided. Consequently, the Editorial Board has lost confidence in the reliability of the overall findings and decided to retract this publication [1], as per MDPI’s retraction policy.

This retraction was approved by the Editor-in-Chief of the *IJMS* journal.

The authors have been informed of this retraction and have formally indicated their disagreement with this decision.
